# LncRNA NUTM2A‐AS1 positively modulates TET1 and HIF‐1A to enhance gastric cancer tumorigenesis and drug resistance by sponging miR‐376a

**DOI:** 10.1002/cam4.3544

**Published:** 2020-10-22

**Authors:** Ji Wang, Ziyang Yu, Jun Wang, Yidan Shen, Junlan Qiu, Zhixiang Zhuang

**Affiliations:** ^1^ Department of Oncology The Second Affiliated Hospital of Soochow University Suzhou China; ^2^ Department of gynaecology and obstetrics The Second Affiliated Hospital of Soochow University Suzhou China; ^3^ Department of General Surgery The Fifth People’s Hospital of Wujiang Suzhou China; ^4^ Department of Oncology and Hematology The Affiliated Suzhou Science and Technology Town Hospital of Nanjing Medical University Suzhou China

**Keywords:** gastric cancer, miR‐376a, NUTM2A‐AS1, PD‐L1, TET1 and HIF‐1A, tumorigenesis and drug resistance

## Abstract

Long noncoding RNA NUTM2A‐AS1 has been shown to be dysregulated in non‐small cell lung carcinoma. To date, it is unclear whether NUTM2A‐AS1 plays a role in gastric cancer progression. The purpose of this study is to elucidate the molecular mechanism of the role of NUTM2A‐AS1 in gastric cancer. mRNA and protein levels were measured by RT‐qPCR and western blot methods. Invasion ability was examined by transwell assay. Cell viability was determined by MTT assay. Dual‐luciferase assay, RNA pull down, and RNA immunoprecipitation were used to confirm direct binding of between miR‐376a and NUTM2A‐AS1 or TET1. Xenografting tumor assay and TCGA analysis showed the contributory role of NUTM2A‐AS1 in vivo and human clinical setting. Our results suggested that NUTM2A‐AS1 promoted cell viability, invasion, and drug resistance of gastric cancer cells, which was largely rescued by miR‐376a. More interestingly, TET1 and HIF‐1A were negatively regulated by miR‐376a. TET1 could interact with HIF‐1A to modulate PD‐L1. Finally, we revealed that PD‐L1 was key to NUTM2A‐AS1‐ and miR‐376a‐mediated tumorigenesis and drug resistance. In summary, our conclusions facilitate us understand the underlying mechanism and develop novel treatment strategy for gastric cancer.

## Graphical abstract

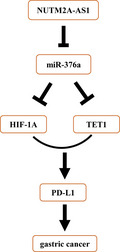

## 1 INTRODUCTION

Gastric cancer (GC) has been shown as the fourth most prevalent cancer type.^1^ It ranks as the second leading cause of cancer‐related mortality around the world.^2^ Five‐year survival rate of GC patient is 5%–20% and the median overall survival is <1 year.^3^, ^4^ To date, chemotherapy (like cisplatin) is used as the first‐line treatment.^5^ Because of multidrug resistance (MDR), advanced GC patients have poor prognosis.^6^

Long noncoding RNAs (LncRNAs) are characterized as ~200 nt long RNAs and have no protein translation capacity.^7^ A large number of evidence show that lncRNAs function in various biological pathways, including transcriptional regulation, cancer progression, and drug resistance.^8^, ^9^ Recently, Amelia Acha‐Sagredo et al reported that NUTM2A‐AS1 was upregulated in non‐small cell lung carcinoma, implying that NUTM2A‐AS1 might exert oncogenic role.^10^ Based on this, it is important to explore the role of NUTM2A‐AS1 in gastric cancer.

To date, it is widely accepted that microRNAs are dysregulated in a variety of cancers and contribute to tumorigenesis and drug resistance.^11^, ^12^ In previous studies, miRNAs could act as oncogenes (e.g., miR‐21) or tumor suppressor genes (e.g., miR‐497) during cancer progression.^13^, ^14^ In gastric cancer, miR‐376a was downregulated and associated with prognosis.^15^ It is hypothesized that miR‐376a may be potential target of NUTM2A‐AS1 in gastric cancer.

DNA methylation is generally believed to involve cancer cell growth and metastasis.^16^ Hao Liu et al. showed that 5hmC was altered in primary gastric cancer.^17^ Ten‐eleven translocation (TET) protein was responsible for conversion of 5mC into 5hmC.^18^ TET1 acted as coactivator of HIF‐1α to modulate gene expression and epithelial‐mesenchymal transition (EMT).^19^ More importantly, TET1 could reduce 5hmC levels and was downregulated in gastric cancer cells.^20^

Here, the purpose of our research is to investigate the specific role of NUTM2A‐AS1 in gastric cancer tumorigenesis and drug resistance. We sought to decipher the mechanism underlying gastric cancer progression, which will advance our understanding of gastric cancer.

## 2 MATERIALS AND METHODS

### 2.1 Cell culture

Human gastric cancer cells (HGC‐27 and SNU‐1) were obtained from Chinese Academy of Sciences Cell Bank of Type Culture Collection. We cultured the cells in Dulbecco's Modified Eagle's Medium (Hyclone) supplemented with 10% of fetal bovine serum and 1% of penicillin/streptomycin. Human gastric mucosal epithelial cell GES‐1 was from Procell Inc. and cultured in RPMI‐1640 medium (Hyclone) supplemented with 10% of fetal bovine serum and 1% of penicillin/streptomycin.

### 2.2 Generation of stable cell lines

shNUTM2A‐AS1‐1 and shNUTM2A‐AS1‐2 stable GC cell lines were generated as standard. Briefly, shRNAs were directly ligated into pLKO.1 vector. Then, 1 µg of total vectors (0.5 µg of pCDH‐shRNA, 0.25 µg of pVSVG, 0.25 µg of pPAX2) were transfected into 293 T cells by lipo2000 for 36–48 h. Lentivirus were collected from supernatant of culture media.

Lentivirus were used to infect GC cells with polybrene for 12 h. After that, the virus‐containing media were replaced with fresh DMEM media and 1 µg/ml of puromycin was utilized to select resistant cells.

### 2.3 Transwell assay

HGC‐27 and SNU‐1 cells (2 × 10^4^ cells) were resuspended in 200 µl of medium without serum. Then, the cells were seeded in upper chamber (Millipore) with 8 μm pore and matrigel‐coated membranes. Then, 300 µl culture medium plus 10% of FBS was added to the bottom chamber as chemoattractant. After 24 h, the non‐invaded cells were removed and invaded cells were stained with 0.05% of crystal violet for 1 h at room temperature. The number of invaded cells were counted under microscope (Olympus).

### 2.4 Dual‐luciferase reporter assay

Wildtype and mutant forms of NUTM2A‐AS1 or 3′‐UTR of TET1 were amplified and subcloned into the pGL3 firefly luciferase vector with double restriction enzymes (KpnI and XhoI). After 48 h of transfection, we examined luciferase activities using the reporter system kit (Promega). Firefly luciferase activity was normalized to Renilla luciferase activity.

### 2.5 Cell Counting kit‐8 (CCK‐8) assay

HGC‐27 and SNU‐1 cells (5 × 10^3^/well) were treated with 1 µmol/L of cisplatin (Selleck) for 0, 24, 48, and 72 h. This assay was performed according to the instructions (Dojindo Molecular Technologies). Cells were digested and resuspended in DMEM medium. Then, cells were plated in 96‐well plates and incubated at 37°C, 5% CO_2_ for a period of 3 days. At the second day, cell growth was stopped by adding 10 µl of CCK‐8 solution (5 mg/ml) to the medium. Finally, OD490 values were measured using a microplate reader (BioTek).

### 2.6 Cell transfection

PD‐L1 cDNA was cloned and inserted into pCMV vector. The recombinant DNA was mixed with lipo2000 (Invitrogen) to transfect into GC cells. In addition, we employed lipofectamine RNAiMAX Reagent (Life Technologies) to bring miRNAs (50 nmol/L) into GC cells. miR‐376a mimic or miR‐376a inhibitor sequences were designed and generated by Shanghai GenePharma.

NC mimic: 5′‑GUACACGCAUCCAAUGAACUCU‑3′;

miR‐376a mimic: 5′‑AUCCGUACCUUAGGUCAUCGAA‑3′;

NC inhibitor: 5′‑CAGUCAUUUACUGUACAUCAA‑3′;

miR‐376a inhibitor: 5′‑UCUAGGUCCAUGCCAUUCAAG‑3′;

### 2.7 Western blot

Protein levels were examined by western blot analysis. In this experiment, ~50 µg of proteins were loaded and separated in 10% of SDS‐PAGE gel. Proteins in SDS‐PAGE gel were transferred to PVDF membrane. PVDF membrane was blocked with 5% of nonfat milk in TBST solution. The proteins were recognized with corresponding primary antibodies for overnight at 4°C and secondary antibodies for 1 h at room temperature. The membrane with proteins was incubated with ECL solution. The signalings were exposed using Bio‐Rad chemiluminescence system. All antibodies were used: anti‐TET1 antibody (MA5‐16312, 1:1000, Invitrogen), anti‐HIF‐1A antibody (20960‐1‐AP, 1:1000, Proteintech), anti‐GAPDH antibody (AF0911‐BP, 1:1000, Affinity Biosciences), anti‐mouse IgG antibody (ab6728, 1:3000, Abcam), anti‐rabbit IgG antibody (ab97051, 1:3000, Abcam).

### 2.8 Reverse transcription‐quantitative PCR (RT‐qPCR)

RNAs were extracted from cells using TRIzol® reagent (Invitrogen). cDNA was synthesized from 1 µg of RNAs using PrimeScript RT kit (Takara Biotechnology). Reaction condition of reverse transcription: 42°C, 30 min; 85°C, 5 s. The cDNAs were diluted with 40 µl of RNase‐free water. Next, 1 µl of cDNA was used for real‐time PCR experiment in 10 µl system with SYBR Green I Master Mix kit (Thermo Fisher Scientific) and primers. The program for PCR on ABI 7500: 94°C for 10 min, 40 cycles of 94°C for 30 s, 60°C for 30 s, and 70°C for 30 s. The relative gene expression was calculated using 2^−ΔΔCT^ method.

The primers were used in real‐time PCR experiments:

NUTM2A‐AS1‐Forward: TACCTCTAGTTCTTCCCGGC;

NUTM2A‐AS1‐Reverse: TTTTGCTTTTCTCCTGGCCC;

MiR‐376a‐Forward: TAAAAGGTAGATTCTCC;

MiR‐376a‐Reverse: GAAAACGTGGATTTTCC;

TET1‐Forward: CAGTGTGTGCTCCTTTTCCC;

TET1‐Reverse: TAGGAGACGTAGACCCACCA;

HIF‐1A‐Forward: TCCAAGAAGCCCTAACGTGT;

HIF‐1A‐Reverse: TGATCGTCTGGCTGCTGTAA;

PD‐L1‐Forward: CCACCACCACCAATTCCAAG;

PD‐L1‐Reverse: TGGCTCCCAGAATTACCAAGT;

GAPDH‐Forward: CAGCCTCAAGATCATCAGCA;

GAPDH‐Reverse: ATGATGTTCTGGAGAGCCCC;

U6‐Forward: AGAAGATTAGCATGGCCCCT;

U6‐Reverse: ATTTGCGTGTCATCCTTGCG.

### 2.9 RNA pull down

lncRNA NUTM2A‐AS1 was labeled with biotin. HGC‐27 cells transfected with indicated vectors were lysed by lysis buffer with RNase inhibitor DEPC. Then, NUTM2A‐AS1‐biotin was incubated with the supernatant of lysis buffer for 4–6 h at 4°C. Streptavidin beads were added to reaction solution at room temperature for 1–2 h. The beads were washed for 2–3 times. Pulled down miR‐376a was examined by RT‐qPCR.

### 2.10 Lymphatic vessel formation assay

HDLEC cells were subject to treatment of conditioned medium (CM) of GC cells on GFR Matrigel diluted with PBS buffer (1:5). Diluted GFR Matrigel solution was used to coat 24‐well plates and incubated at 37°C for at least 5 h. HDLEC cells were seeded in DMEM medium at density of ~40,000 cells/well. We used inverted microscope to observe tube length.

### 2.11 Trypan blue staining

LD50 was determined by this assay, 0.4% of trypan blue solution (Beyotime) was added and the cells were counted under Countess Automated Cell Counter (Invitrogen). Dead cells were defined as cells could not exclude the dye.

### 2.12 Xenograft experiment

In this experiment, eight NOD‐SCID mice (four mice per group) were used. Mice were injected subcutaneously with a million of HGC‐27 cells. After 4 weeks, the mice were sacrificed and tumors were excised. The animal protocol was approved by the Animal Welfare Committee of The Second Affiliated Hospital of Soochow University. Tumor volume = 0.5 × length × width^2^.

### 2.13 The Cancer Genome Atlas (TCGA)

The gene expression datasets of stomach adenocarcinoma were downloaded from TCGA datasets (211 normal tissues and 408 tumors). Gene expression box plot, Stage plot, and Kaplan–Meier plot analyses were performed by GraphPad Prism software.

### 2.14 RNA immunoprecipitation (RIP) assay

RIP was performed using a Magna RNA Immunoprecipitation kit (Millipore). Lysates of HGC‐27 cells (1 × 10^7^) were incubated with protein A/G magnetic beads conjugated with normal mouse IgG or anti‐Ago2 antibody. The detailed protocol was referenced by manufacturers’ instructions. The immunoprecipitated NUTM2A‐AS1 and miR‐376a were detected by RT‐qPCR.

### 2.15 Isolation of cytoplasmic and nuclear RNA

Cytoplasmic and nuclear RNAs of HGC‐27 and SNU‐1 cells were extracted and purified using PARIS™ Kit (AM1921, Invitrogen) according to the manufacturer's instructions.

### 2.16 Northern blot analysis

In this experiment, 40 μg of RNA was separated by formaldehyde gel electrophoresis. Then, the RNA‐contained gel was transferred to a Biodyne Nylon membrane that was fixed by UV crosslinking. After prehybridization in Ultrahyb buffer (Ambion) at 62°C for 1 h, the membrane was hybridized in Ultrahyb buffer with digoxin‐labeled probes for NUTM2A‐AS1 at 62°C overnight. The membrane was then incubated with anti‐DIG‐biotin antibody (BOSTER Biological Technology) for 2 h at room temperature. Then, the membrane was incubated with HRP‐conjugated Streptavidin for 30 min at room temperature. Finally, the expression of NUTM2A‐AS1 was detected. NUTM2A‐AS1 probe for northern blot: AGGAGAAAAGCAAAACCATATTCCT.

### 2.17 Statistical analysis

GraphPad Prism 8.0 was utilized to perform statistical analysis. The data of three replicates were expressed as mean ± standard deviation (SD). Comparisons of two groups were analyzed using non‐paired Student's *t* tests. Three or more groups comparisons were done by ANOVA (Tukey's post hoc test). In all results, *p* < 0.05 was considered as statistically significant.

## 3 RESULTS

### 3.1 NUTM2A‐AS1 knockdown‐attenuated GC cell tumorigenesis and drug resistance

**Figure 1:**
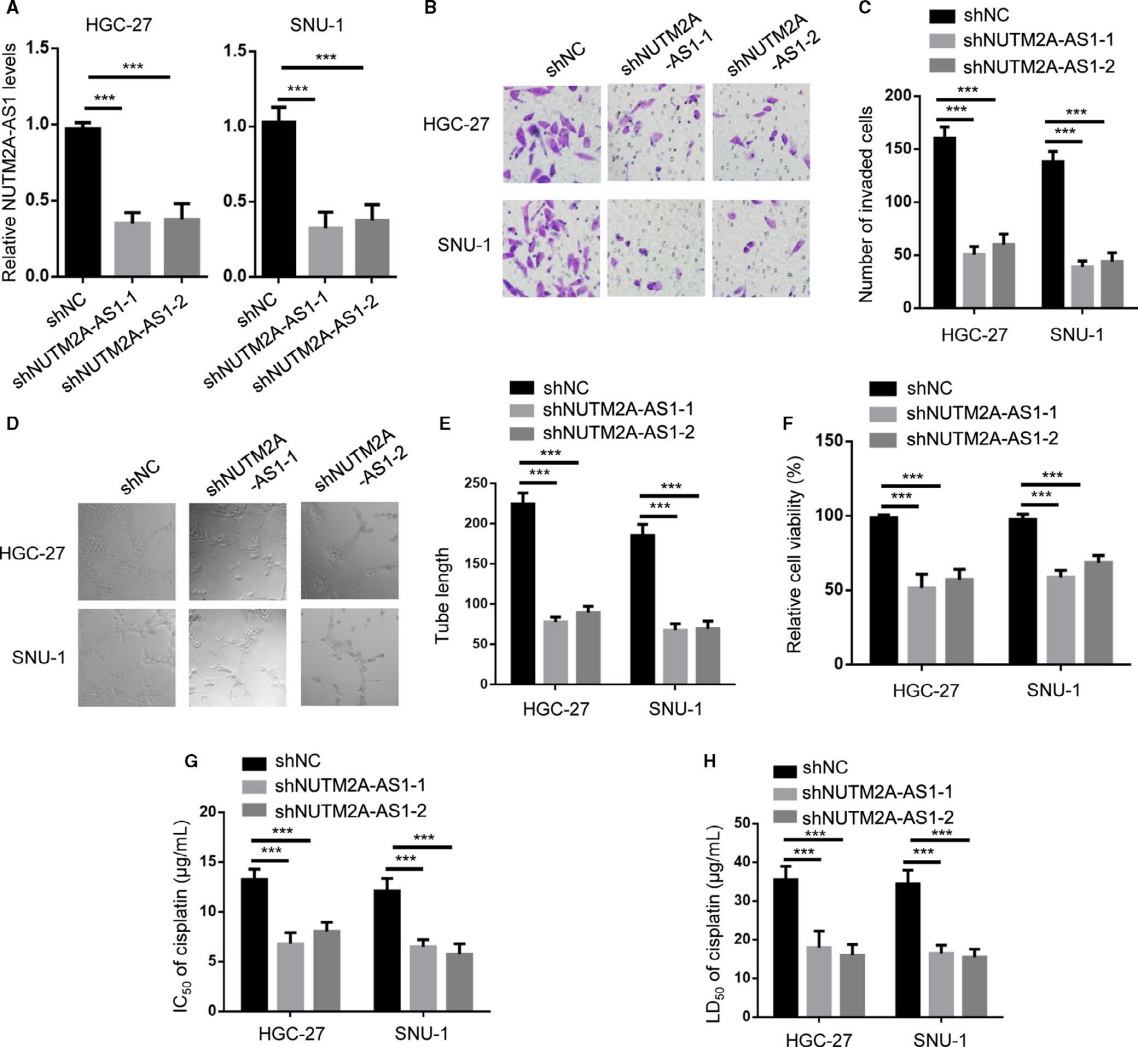
NUTM2A‐AS1 knockdown‐attenuated GC cell tumorigenesis and drug resistance. (A) RT‐qPCR showed NUTM2A‐AS1 expression levels in HGC‐27 and SNU‐1 cells stably expressing negative control (shNC), shNUTM2A‐AS1‐1, shNUTM2A‐AS1‐2. ***p < 0.001. (B and C) Transwell assay showed invasion of HGC‐27 and SNU‐1 cells stably expressing shNC, shNUTM2A‐AS1‐1, shNUTM2A‐AS1‐2. ***p < 0.001. (D and E) Lymphatic vessel formed by HDLEC cells cultured with conditioned medium (CM) of HGC‐27 and SNU‐1 cells transfected with shNC, shNUTM2A‐AS1‐1, shNUTM2A‐AS1‐2. ***p < 0.001. (F) CCK‐8 assay showed cell viability of HGC‐27 and SNU‐1 cells stably expressing shNC, shNUTM2A‐AS1‐1, shNUTM2A‐AS1‐2. ***p < 0.001. (G) IC_50_ measurement of cisplatin‐treated HGC‐27 and SNU‐1 cells stably expressing shNC, shNUTM2A‐AS1‐1, shNUTM2A‐AS1‐2. ***p < 0.001. (H) LD_50_ measurement of cisplatin‐treated HGC‐27 and SNU‐1 cells stably expressing shNC, shNUTM2A‐AS1‐1, shNUTM2A‐AS1‐2. ***p < 0.001

To explore the biological role of NUTM2A‐AS1, we generated NUTM2A‐AS1 knockdown stable cell lines (HGC‐27, SNU‐1) by shRNAs (Figure 1A). Transwell assay showed less invaded cell number of HGC‐27 and SNU‐1 cells depleted of NUTM2A‐AS1 (Figure 1B,C). Moreover, NUTM2A‐AS1 knockdown significantly impaired lymphatic vessel formation ability of HDLEC cells incubated with CM of HGC‐27 and SNU‐1 (Figure 1D,E). Cell viability of HGC‐27 and SNU‐1 cells were attenuated when the cells were introduced with shNUTM2A‐AS1 compared to shNC (Figure 1F). IC50 values of HGC‐27 and SNU‐1 cells transfected with shNUTM2A‐AS1 were reduced by ~40% compared to shNC group cells (Figure 1G). LD50 of cisplatin in NUTM2A‐AS1 knockdown HGC‐27 and SNU‐1 cells were lower than that of shNC cells (Figure 1H). These results suggested that NUTM2A‐AS1 played oncogenic role in gastric cancer tumorigenesis and drug resistance.

### 3.2 NUTM2A‐AS1 depletion inhibited tumor growth of GC cells in animal model and clinical setting

**Figure 2:**
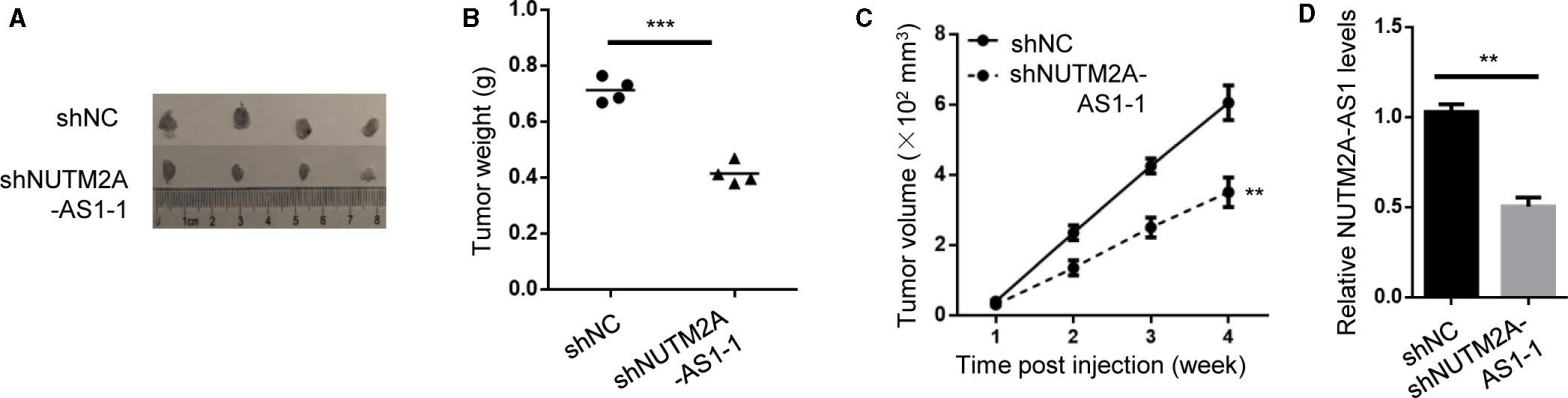
NUTM2A‐AS1 depletion inhibited tumor growth of GC cells in vivo. (A) Xenografting tumor assay showed tumor growth of HGC‐27 cells stably expressing shNC, shNUTM2A‐AS1‐1 in immunodeficient mice. *n* = 4. (B and C) Tumor weights (B) and volumes (C) were measured. **p < 0.01; ***p < 0.001. (D) RT‐qPCR showed NUTM2A‐AS1 expression levels in tumors formed by HGC‐27 cells stably expressing shNC, shNUTM2A‐AS1‐1. **p < 0.01

Next, we asked if NUTM2A‐AS1 exerted the oncogenic role in animal model. shNC or shNUTM2A‐AS1‐expressed HGC‐27 cells were subcutaneously implanted under skin of immunodeficient mice (Figure 2A). Tumor weight and volume results indicated that shNUTM2A‐AS1 tumors were remarkably smaller than shNC tumors (Figure 2B,C). RT‐qPCR data confirmed NUTM2A‐AS1 was downregulated in shNUTM2A‐AS1 tumors (Figure 2D).

**Figure 3:**
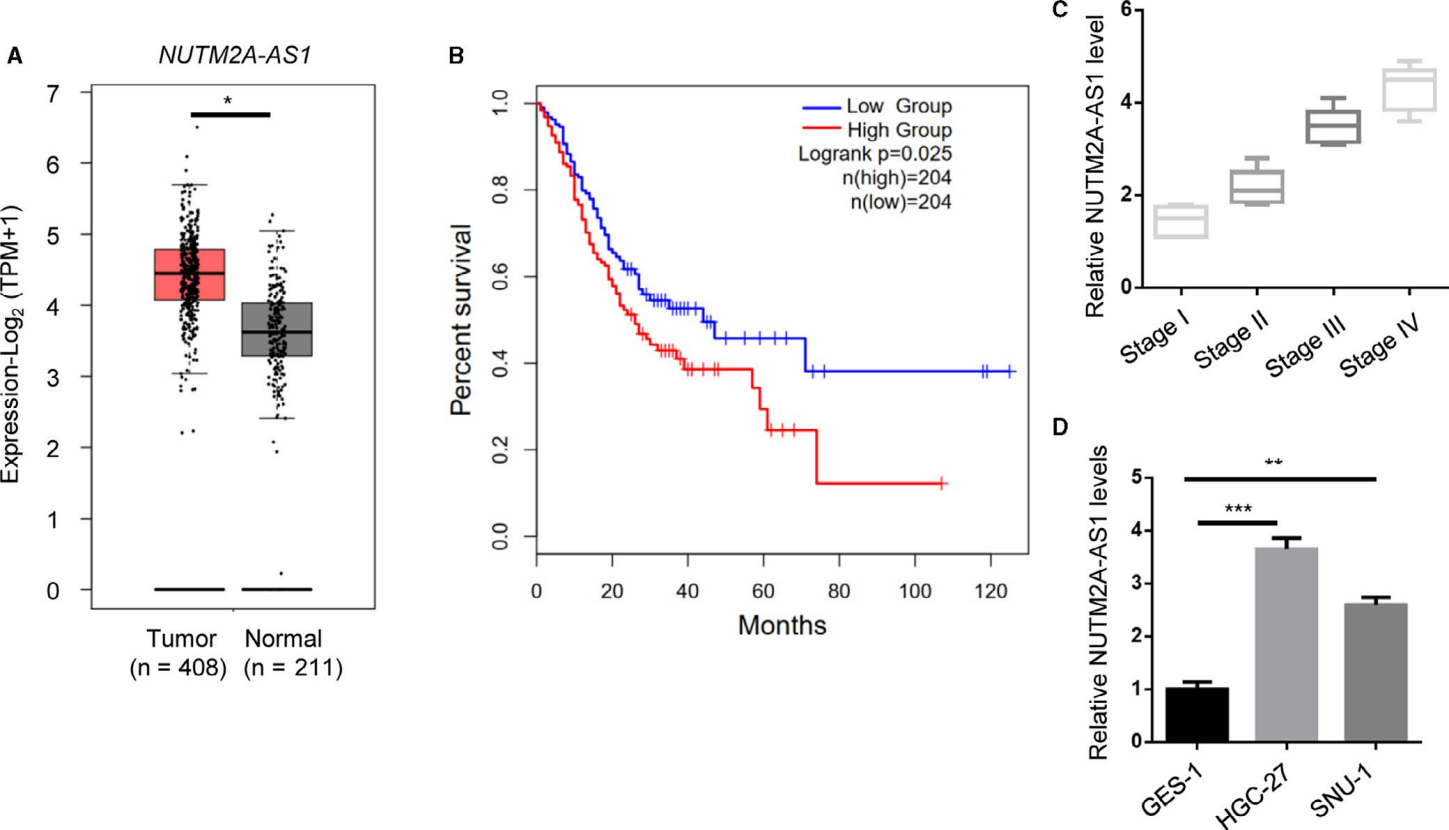
NUTM2A‐AS1 was upregulated and associated with poor prognosis of GC patients. (A) NUTM2A‐AS1 levels of normal tissues (*n* = 211) and tumors (*n* = 408) were analyzed in TCGA datasets of GC patients. *p < 0.05. (B) Kaplan–Meier analysis showed overall survival (OS) of GC patients by low group (*n* = 204) and high group (*n* = 204). *p* = 0.0025. (C) Stage plot analysis showed higher expression of NUTM2A‐AS1 in advanced stage (stage III and IV) of GC patients. (D) NUTM2A‐AS1 levels of normal gastric mucosal epithelial cell GES‐1 and GC cell lines (HGC‐27 and SNU‐1 cells) were examined by RT‐qPCR. **p < 0.01; ***p < 0.001

In addition to animal model, we also sought to investigate whether NUTM2A‐AS1 acted as oncogene in human clinical tissues. We found that NUTM2A‐AS1 was relatively elevated in gastric tumors (Figure 3A). Moreover, patients with high level of NUTM2A‐AS1 lived for a shorter time period (Figure 3B). Advanced stage of tumors displayed higher level of NUTM2A‐AS1, compared with primary stages of tumors (Figure 3C). Besides, we carried out RT‐qPCR to examine NUTM2A‐AS1 levels in human gastric mucosal epithelial cell GES‐1 and gastric cancer cells HGC‐27 and SNU‐1. Expectedly, higher NUTM2A‐AS1 expressions were detected in HGC‐27 and SNU‐1 cells in relative to GES‐1 cell (Figure 3D). In conclusion, our data revealed that NUTM2A‐AS1 contributed to gastric cancer progression in animal and clinical environment.

### 3.3 miR‐376a‐mediated NUTM2A‐AS1 knockdown‐attenuated GC cell tumorigenesis and drug resistance

**Figure 4:**
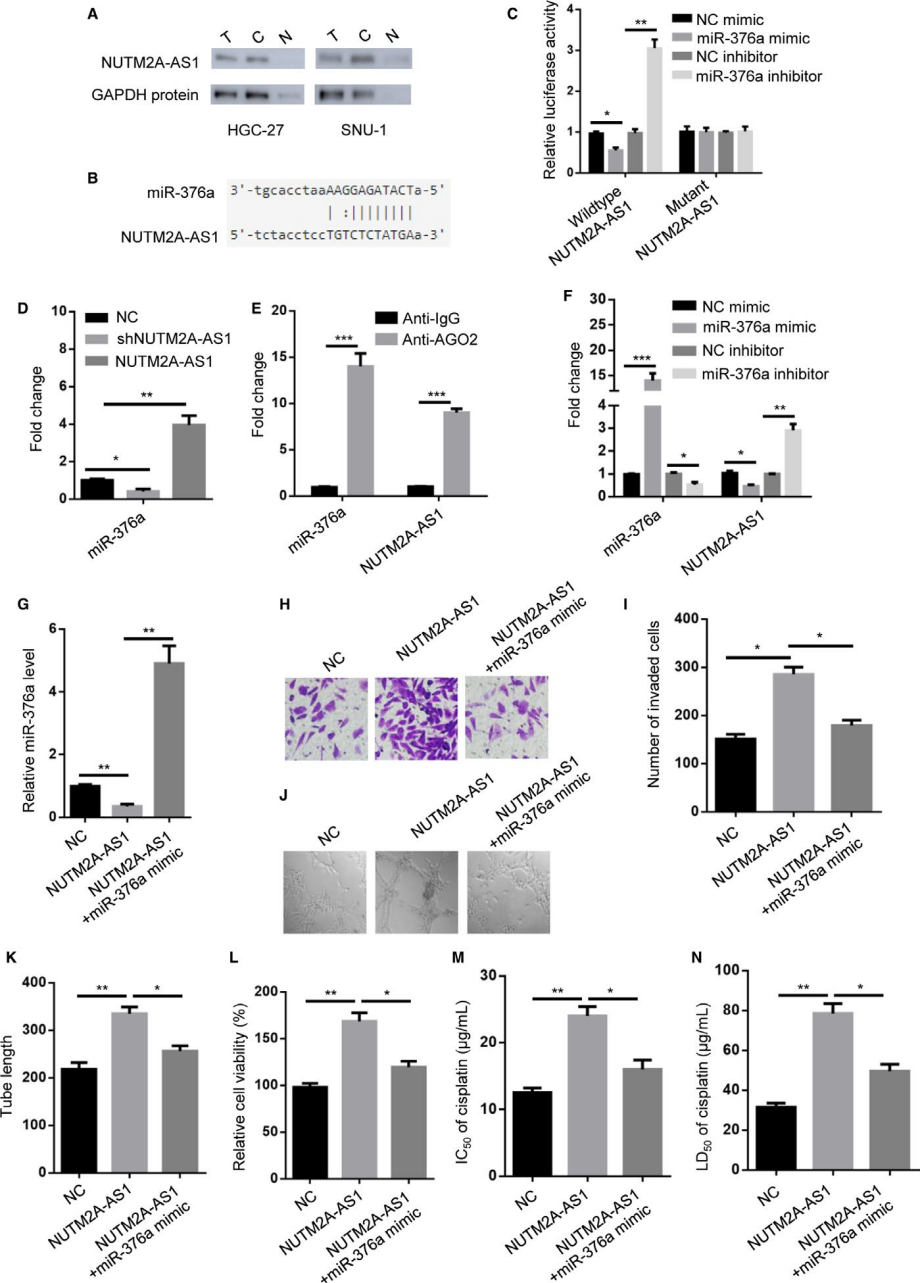
miR‐376a‐mediated NUTM2A‐AS1 knockdown‐attenuated GC cell tumorigenesis and drug resistance. (A) Northern blot and western blot analysis showed NUTM2A‐AS1 and GAPDH levels in total (T), cytoplasmic (C), and nuclear (N) fractions. GAPDH served as loading control. (B) Bioinformatics prediction (StarBase v2.0 database) of putative binding site at NUTM2A‐AS1 by miR‐376a. (C) Luciferase activity was measured in HGC‐27 cells transfected with pGL3‐NUTM2A‐AS1 plus NC mimic, miR‐376a mimic, NC inhibitor, miR‐376a inhibitor. *p < 0.05; **p < 0.01. (D) RNA pull down was performed to detect miR‐376a binding by NUTM2A‐AS1. *p < 0.05; **p < 0.01. (E) RIP assay showed that NUTM2A‐AS1 bound to miR‐376a. ***p < 0.001. (F) RT‐qPCR showed miR‐376a and NUTM2A‐AS1 expression levels in HGC‐27 cells transfected with NC mimic, miR‐376a mimic, NC inhibitor, miR‐376a inhibitor. *p < 0.05; **p < 0.01; ***p < 0.001. (G) RT‐qPCR showed miR‐376a levels in HGC‐27 cells stably expressing pcDNA3.1, pcDNA3.1‐NUTM2A‐AS1, pcDNA3.1‐NUTM2A‐AS1 plus miR‐376a mimic. **p < 0.01. (H and I) Transwell assay showed invasion of HGC‐27 cells stably expressing pcDNA3.1, pcDNA3.1‐NUTM2A‐AS1, pcDNA3.1‐NUTM2A‐AS1 plus miR‐376a mimic. *p < 0.05. (J and K) Lymphatic tube formation showed tube length of HDLEC cells incubated with conditioned medium of HGC‐27 cells stably expressing pcDNA3.1, pcDNA3.1‐NUTM2A‐AS1, pcDNA3.1‐NUTM2A‐AS1 plus miR‐376a mimic. *p < 0.05; **p < 0.01. (L) CCK‐8 assay showed cell viability of HGC‐27 cells stably expressing pcDNA3.1, pcDNA3.1‐NUTM2A‐AS1, pcDNA3.1‐NUTM2A‐AS1 plus miR‐376a mimic. *p < 0.05; **p < 0.01. (M) IC_50_ measurement of cisplatin‐treated HGC‐27 cells stably expressing pcDNA3.1, pcDNA3.1‐NUTM2A‐AS1, pcDNA3.1‐NUTM2A‐AS1 plus miR‐376a mimic. *p < 0.05; **p < 0.01. (N) LD_50_ measurement of cisplatin‐treated HGC‐27 cells stably expressing pcDNA3.1, pcDNA3.1‐NUTM2A‐AS1, pcDNA3.1‐NUTM2A‐AS1 plus miR‐376a mimic. *p < 0.05; **p < 0.01

To search for molecules involved in NUTM2A‐AS1‐regulated functions in gastric cancer, we first sought to pinpoint the cellular location of NUTM2A‐AS1. Northern blot result showed that NUTM2A‐AS1 mainly located in the cytoplasmic fraction (Figure 4A). Combined with previous reports, we propose that miRNAs are our first‐choice among various molecular targets. Bioinformatics result identified a number of miRNAs as putative targets for NUTM2A‐AS1. However, we found that only miR‐376a was reported to be associated with gastric cancer and the underlying mechanism remained completely unclear, which prompted us to further investigate the miR‐376a in our study (Figure 4B). Hence, we attempt to determine the role of miR‐376a in gastric cancer tumorigenesis and drug resistance. First of all, luciferase reporter assay and RNA pull down experiment were carried out to show direct interaction between NUTM2A‐AS1 and miR‐376a (Figure 4C,D). Besides, RNA immunoprecipitation experiment was performed to confirm that both NUTM2A‐AS1 and miR‐376a existed in Ago2 complex (Figure 4E). More importantly, we found that miR‐376a inhibited NUTM2A‐AS1 expression in HGC‐27 cells (Figure 4F).

We transfected miR‐376a mimic into NUTM2A‐AS1‐overexpressed HGC‐27 cells (Figure 4G). The results of transwell assay displayed that NUTM2A‐AS1 overexpression led to enhanced invasion of HGC‐27 cells. miR‐376a mimic largely suppressed NUTM2A‐AS1‐modulated cell invasion (Figure 4H,I). Furthermore, we observed that miR‐376a mimic rescued lymphatic vessel formation ability of HDLEC cells co‐incubated with CM of NUTM2A‐AS1‐overexpressed HGC‐27 cells (Figure 4J,K). Likewise, cell viability assay showed that NUTM2A‐AS1 promoted cell viability of HGC‐27 cells, which was reverted by miR‐376a mimic (Figure 4L). IC50 value of HGC‐27 cells treated with cisplatin was increased by NUTM2A‐AS1 overexpression, which was reduced by miR‐376a mimic (Figure 4M). miR‐376a mimic decreased LD50 of cisplatin in NUTM2A‐AS1‐overexpressed HGC‐27 cells (Figure 4N). Taken together, miR‐376a was critical for NUTM2A‐AS1‐induced gastric cancer tumorigenesis and drug resistance.

### 3.4 miR‐376a targets TET1 and HIF1A

**Figure 5:**
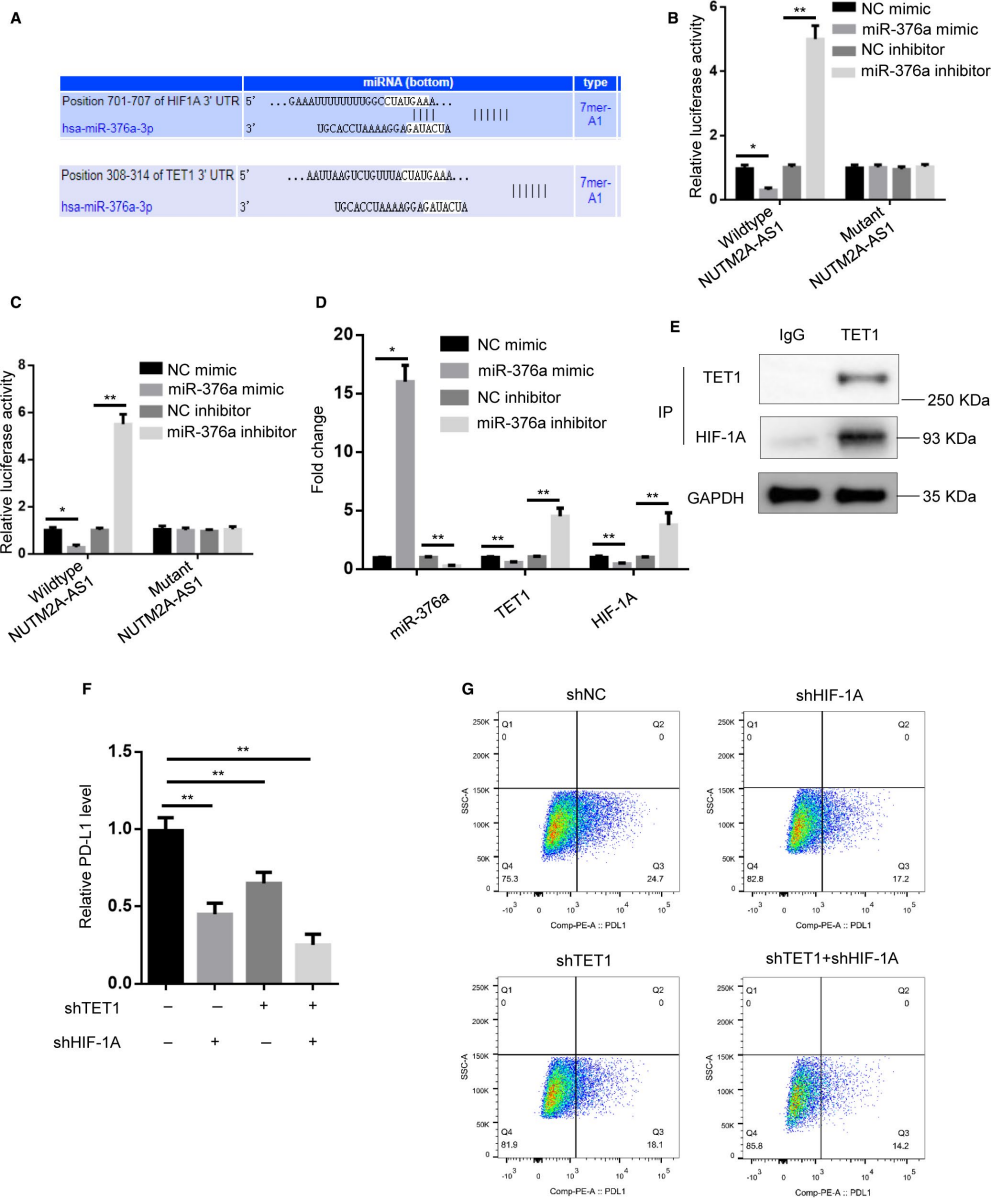
miR‐376a directly targeted TET1 and HIF1A. (A) Bioinformatics prediction (TargetScan database) of putative binding site at 3’‐UTRs of TET1 and HIF‐1A by miR‐376a. (B and C) Luciferase activity was measured in HGC‐27 cells transfected with HIF‐1A or TET1 3’‐UTR‐fused pGL3 vector plus NC mimic, miR‐376a mimic, NC inhibitor, miR‐376a inhibitor. *p < 0.05; **p < 0.01. (D) RT‐qPCR showed miR‐376a, TET1, HIF‐1A expression levels in HGC‐27 cells transfected with NC mimic, miR‐376a mimic, NC inhibitor, miR‐376a inhibitor. *p < 0.05; **p < 0.01. (E) Co‐immunoprecipitation showed interaction of TET1 with HIF‐1A in HGC‐27 cells. IgG served as negative control. (F) RT‐qPCR showed PD‐L1 mRNA levels in HGC‐27 cells transfected with shNC, shHIF‐1A, shTET1, shHIF‐1A plus shTET1. **p < 0.01. (G) Flow cytometry analysis showed PD‐L1 protein levels in HGC‐27 cells transfected with shNC, shHIF‐1A, shTET1, shHIF‐1A plus shTET1

To identify the downstream effectors of miR‐376a in gastric cancer cells, we used bioinformatics software to find out the potential targets of miR‐376a. Among them, an epigenetic regulator TET1 and HIF‐1A were evidently regulated (Figure 5A), which could bridge epigenetic mechanism with NUTM2A‐AS1/miR‐376a‐regulated gastric cancer. Dual‐luciferase reporter assay demonstrated that miR‐376a bound 3’‐UTR regions of TET1 and HIF‐1A (Figure 5B,C). RT‐qPCR results suggested that miR‐376a suppressed the expression levels of TET1 and HIF‐1A (Figure 5D).

### 3.5 TET1 interacts with HIF‐1A to regulate PD‐L1

CO‐IP experiment showed that TET1 could endogenously interact with HIF‐1A in HGC‐27 cells (Figure 5E). Interestingly, RT‐qPCR results indicated that HIF‐1A‐enhanced PD‐L1 expression could be abrogated by TET1 knockdown (Figure 5F). To validate this result, we utilized flow cytometry experiment to examine PD‐L1 level of HGC‐27 and SNU‐1 cells. The data suggested that PD‐L1 protein expression pattern was similar to that of RT‐qPCR (Figure 5G).

### 3.6 PD‐L1 partially rescued NUTM2A‐AS1‐ and miR‐376a‐regulated GC cell tumorigenesis and drug resistance

**Figure 6:**
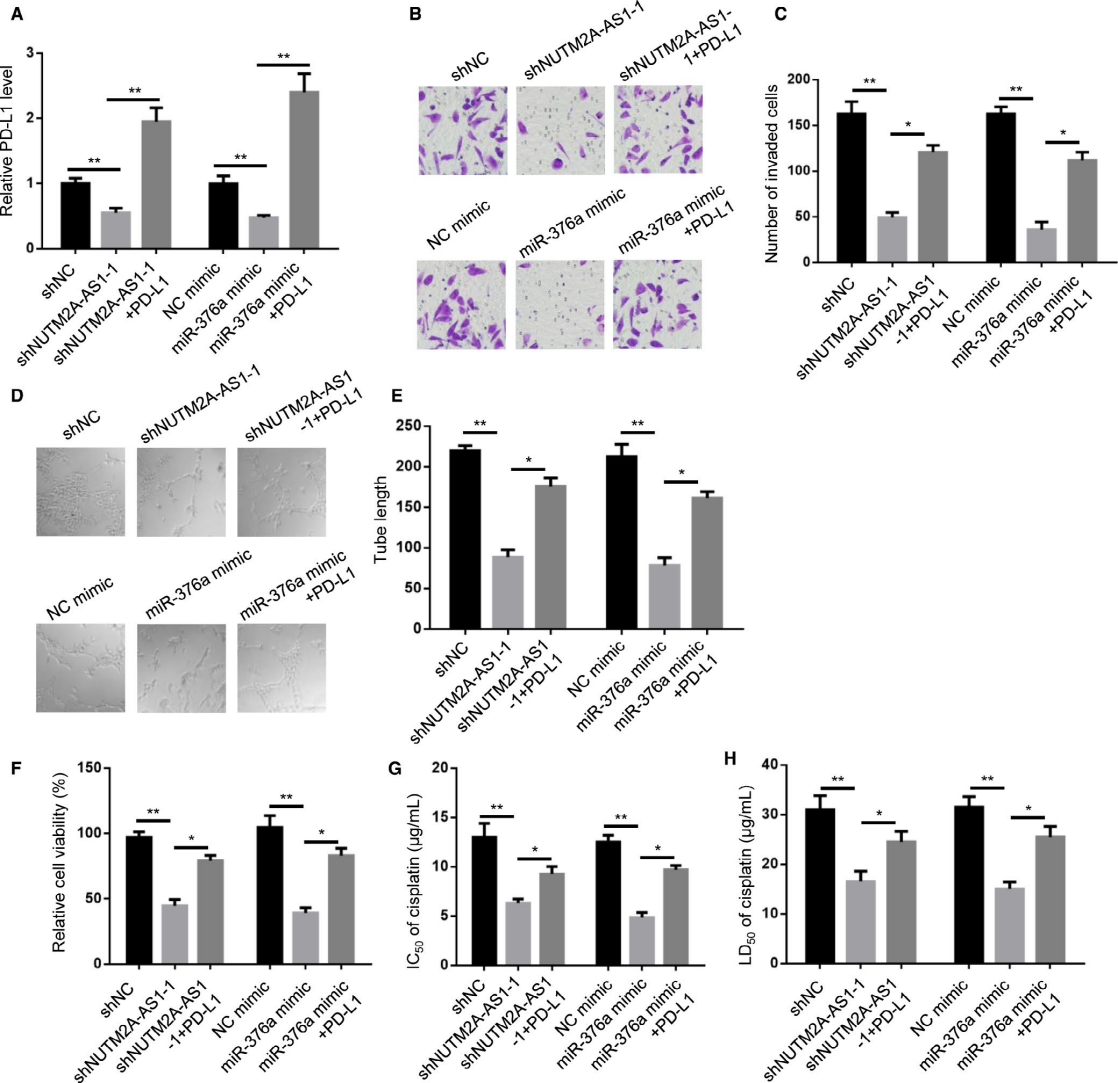
PD‐L1 partially rescued NUTM2A‐AS1‐ and miR‐376a‐regulated GC cell tumorigenesis and drug resistance. (A) RT‐qPCR showed PD‐L1 expression levels in HGC‐27 cells stably expressing shNC, shNUTM2A‐AS1‐1, shNUTM2A‐AS1‐1 plus PD‐L1, NC mimic, miR‐376a mimic, miR‐376a mimic plus PD‐L1. **p < 0.01. (B and C) Transwell assay showed invasion of HGC‐27 cells stably expressing shNC, shNUTM2A‐AS1‐1, shNUTM2A‐AS1‐1 plus PD‐L1, NC mimic, miR‐376a mimic, miR‐376a mimic plus PD‐L1. *p < 0.05; **p < 0.01. (D and E) Lymphatic vessel formed by HDLEC cells cultured with conditioned medium of HGC‐27 cells stably expressing shNC, shNUTM2A‐AS1‐1, shNUTM2A‐AS1‐1 plus PD‐L1, NC mimic, miR‐376a mimic, miR‐376a mimic plus PD‐L1. *p < 0.05; **p < 0.01. (F) CCK‐8 assay showed cell viability of HGC‐27 cells stably expressing shNC, shNUTM2A‐AS1‐1, shNUTM2A‐AS1‐1 plus PD‐L1, NC mimic, miR‐376a mimic, miR‐376a mimic plus PD‐L1. *p < 0.05; **p < 0.01. (G) IC_50_ measurement of cisplatin‐treated HGC‐27 cells stably expressing shNC, shNUTM2A‐AS1‐1, shNUTM2A‐AS1‐1 plus PD‐L1, NC mimic, miR‐376a mimic, miR‐376a mimic plus PD‐L1. *p < 0.05; **p < 0.01. (H) LD_50_ measurement of cisplatin‐treated HGC‐27 cells stably expressing shNC, shNUTM2A‐AS1‐1, shNUTM2A‐AS1‐1 plus PD‐L1, NC mimic, miR‐376a mimic, miR‐376a mimic plus PD‐L1. *p < 0.05; **p < 0.01

To investigate the role of PD‐L1 in NUTM2A‐AS1‐ and miR‐376a‐regulated gastric cancer tumorigenesis and drug resistance, we overexpressed PD‐L1 in shNUTM2A‐AS1‐1‐ and miR‐376a mimic‐expressed HGC‐27 cells (Figure 6A). Transwell assay result showed that PD‐L1 rescued NUTM2A‐AS1 knockdown‐ and miR‐376a mimic‐attenuated invasion of HGC‐27 cells (Figure 6B,C). Lymphatic vessel formation assay showed that PD‐L1 overexpression resulted in increased tube length of shNUTM2A‐AS1‐1‐ and miR‐376a mimic‐expressed HGC‐27 cells (Figure 6D,E). In parallel, PD‐L1 restored NUTM2A‐AS1 knockdown‐ and miR‐376a mimic‐attenuated cell viability of HGC‐27 cells (Figure 6F). Substantial increase in IC50 value was observed in PD‐L1‐overexpressed cells (Figure 6G). LD50 of cisplatin was also elevated in PD‐L1‐overexpressed HGC‐27 cells (Figure 6H). To sum up, PD‐L1 served as a key effector for NUTM2A‐AS1‐ and miR‐376a‐modulated gastric cancer tumorigenesis and drug resistance.

## 4 DISCUSSION

**Figure 7:**
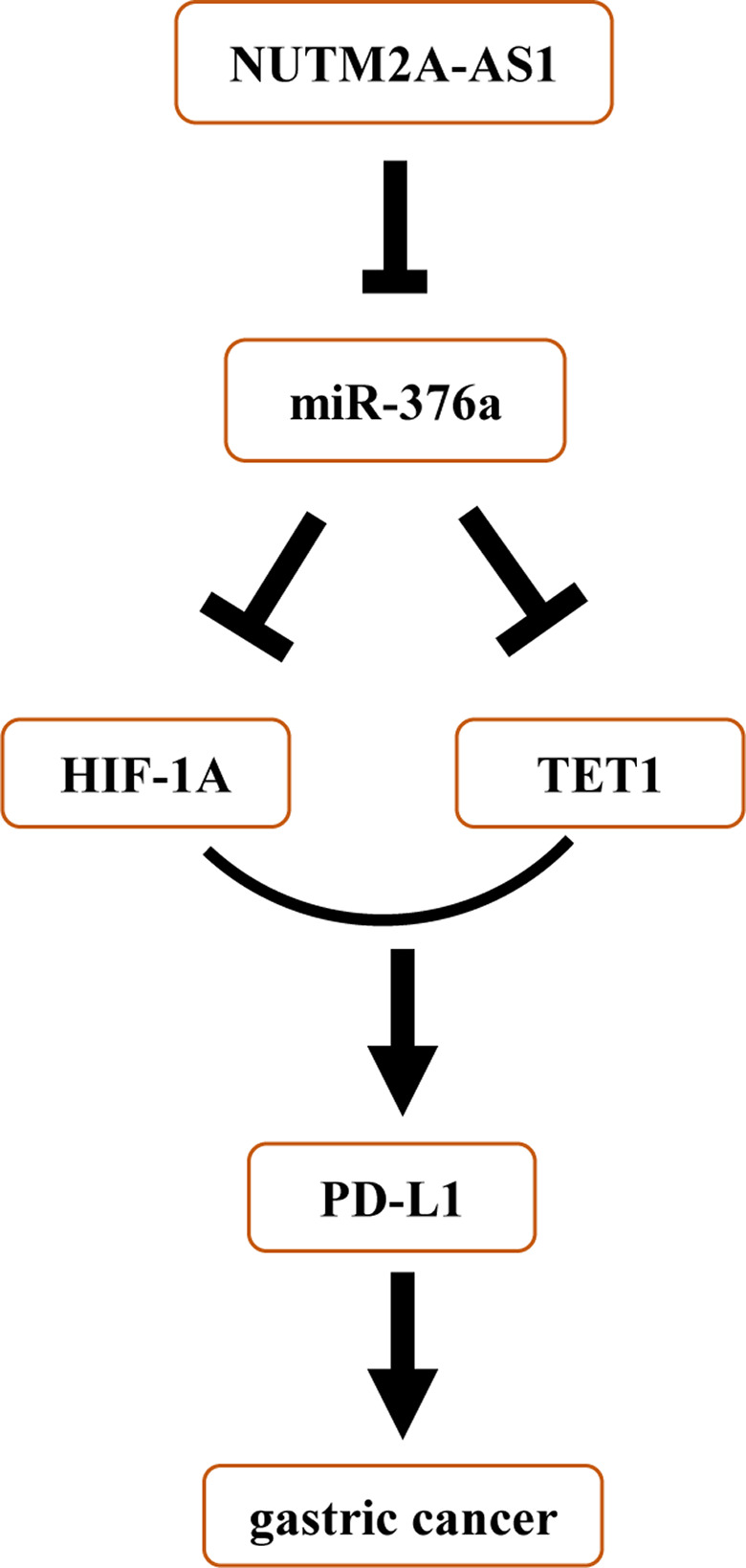
Schematic diagram showed the mechanism by which NUTM2A‐AS1 played its role in gastric cancer malignancy.

In this study, we focused on investigating the role of NUTM2A‐AS1 in gastric cancer (Figure 7). miR‐376a was a newly identified miRNA interacting with NUTM2A‐AS1 and had key effect on NUTM2A‐AS1‐induced tumorigenesis and drug resistance of GC. Epigenetic regulator TET1 directly bound HIF‐1A to regulate immune checkpoint PD‐L1. All these conclusions related epigenetic treatment and immunotherapy to gastric cancer.

At present, surgical resection is the first‐choice for early‐stage GC treatment. However, gastric cancer is prone to be resistant to chemotherapy drugs.^21^ Recently, combination treatment of chemotherapy and molecularly targeted agents has been proposed to be used in advanced stage of GC.^22^ Although big progress has been achieved, intrinsically heterogeneous trait of GC leads to poor prognosis.^23^ More interestingly, immunotherapy using antibodies targeting CTLA‐4 and PD‐L1/PD‐1 (e.g., pembrolizumab, durvalumab, atezolizumab, and avelumab) have shown promising in clinical trials. In 2017, pembrolizumab was approved by FDA for metastatic gastric cancer as the first immune checkpoint inhibitor drug.^24^ In our study, we presented that PD‐L1 was important for NUTM2A‐AS1‐mediated gastric cancer progression.

So far, a number of evidence show that lncRNAs are involved in tumor progression and cisplatin resistance of gastric cancer via various mechanisms.^25^, ^26^, ^27^, ^28^ NUTM2A‐AS1 has not been studied in cancer, including gastric cancer. Our group, for the first time, prove that NUTM2A‐AS1 accelerates tumor invasion, angiogenesis, and cisplatin resistance at cellular and animal level. In previous reports, lncRNAs acted as ceRNA of miRNAs by its miRNA‐recognition elements (MREs).^29^ Among the prediction results, miR‐376a directly bound NUTM2A‐AS1 and functioned as tumor suppressor in gastric cancer.

Epigenetic regulation is implicated in biological processes in mammals.^30^ In tumors, transcriptions of tumor suppressors are commonly inhibited by promoter hypermethylation.^31^ It is of our interest that TET1 is a downstream target of miR‐376a. TET1 serves as tumor suppressor in a variety of cancer types, such as colon,^32^ ovarian,^33^ pancreatic,^34^ and gastric cancer.^35^ Generally, TET1 exerts the roles through its enzymatic activity toward converting 5mC into 5hmC, 5fC and 5caC.^18^ Unexpectedly, TET1 interacted with HIF‐1A to modulate PD‐L1 level in its enzymatic activity‐independent manner.^19^ One of the possible reason was that TET1 recruited classical transcription factors (e.g., HIF‐1A) to influence gene expression.

Programed cell death‐1 (PD‐1) and its ligand PD‐L1 are well‐known immune checkpoint signaling.^36^ PD‐L1 is upregulated and associated with immune evasion of cancer cells.^37^ Muhammad Zaeem Noman et al showed that PD‐L1 was direct target of HIF‐1A.^38^ Consistent with this, we revealed that TET1/HIF‐1A complex positively regulated PD‐L1. Moreover, PD‐L1 overexpression contributed to the function of NUTM2A‐AS1/miR‐376a for malignancy of gastric cancer. Hence, it might be possible to treat gastric cancer by combination of cisplatin and anti‐PD‐L1 monoclonal antibody drug.

In summary, our research provides another strategy for GC diagnosis and treatment by targeting NUTM2A‐AS1/miR‐376a/TET1/PD‐L1 axis. Combinatorial utilization of traditional chemotherapy and immunotherapy will be more effective than single treatment for GC.

## CONFLICT OF INTERESTS

The authors declare that there are no conflict of interests.

## AUTHOR CONTRIBUTIONS

Ji Wang and Zhixiang Zhuang: designed and performed experiments; Ji Wang, Ziyang Yu, and Jun Wang: performed most experiments and data analysis; Yidan Shen: prepared figures; Junlan Qiu: statistical analysis and helpful suggestions; Zhixiang Zhuang: write the manuscript.

## Data Availability

All the data generated and/or analyzed during the current study are available from the corresponding author on reasonable request.

## References

[1] Rawla P , Barsouk A . Epidemiology of gastric cancer: global trends, risk factors and prevention. Gastroenterol Rev. 2019;14(1):26‐38. 10.5114/pg.2018.80001 PMC6444111 30944675

[2] Cavatorta O , Scida S , Miraglia C , et al. Epidemiology of gastric cancer and risk factors. Acta Bio‐Medica. 2018;89(8‐S):82‐87. 30561423 10.23750/abm.v89i8-S.7966 PMC6502220

[3] Karimi P , Islami F , Anandasabapathy S , Freedman ND , Kamangar F . Gastric cancer: descriptive epidemiology, risk factors, screening, and prevention. Cancer Epidemiol Biomark Prev. 2014;23(5):700‐713. 10.1158/1055-9965.EPI-13-1057 PMC4019373 24618998

[4] Van Cutsem E , Sagaert X , Topal B , Haustermans K , Prenen H . Gastric cancer. Lancet. 2016;388(10060):2654‐2664. 27156933 10.1016/S0140-6736(16)30354-3

[5] Charalampakis N , Economopoulou P , Kotsantis I , et al. Medical management of gastric cancer: a 2017 update. Cancer Med. 2018;7(1):123‐133. 29239137 10.1002/cam4.1274 PMC5773977

[6] Huang H , Tang J , Zhang L , Bu Y , Zhang X . miR‐874 regulates multiple‐drug resistance in gastric cancer by targeting ATG16L1. Int J Oncol. 2018;53(6):2769‐2779. 30320370 10.3892/ijo.2018.4593

[7] Zhang Z , Qian W , Wang S , et al. Analysis of lncRNA‐associated ceRNA network reveals potential lncRNA biomarkers in human colon adenocarcinoma. Cell Physiol Biochem. 2018;49(5):1778‐1791. 30231249 10.1159/000493623

[8] Fang Q , Chen X , Zhi X . Long non‐coding RNA (LncRNA) urothelial carcinoma associated 1 (UCA1) increases multi‐drug resistance of gastric cancer via downregulating miR‐27b. Med Sci Monit. 2016;22:3506‐3513. 27694794 10.12659/MSM.900688 PMC5051552

[9] Yang Y , Jiang C , Yang Y , et al. Silencing of LncRNA‐HOTAIR decreases drug resistance of Non‐Small Cell Lung Cancer cells by inactivating autophagy via suppressing the phosphorylation of ULK1. Biochem Biophys Res Comm. 2018;497(4):1003‐1010. 29470986 10.1016/j.bbrc.2018.02.141

[10] Acha‐Sagredo A , Uko B , Pantazi P , et al. Long non‐coding RNA dysregulation is a frequent event in non‐small cell lung carcinoma pathogenesis. Br J Cancer. 2020;122(7):1050‐1058. 32020063 10.1038/s41416-020-0742-9 PMC7109049

[11] Dehghanzadeh R , Jadidi‐Niaragh F , Gharibi T , Yousefi M . MicroRNA‐induced drug resistance in gastric cancer. Biomed Pharmacother. 2015;74:191‐199. 26349984 10.1016/j.biopha.2015.08.009

[12] Zheng P , Chen L , Yuan X , et al. Exosomal transfer of tumor‐associated macrophage‐derived miR‐21 confers cisplatin resistance in gastric cancer cells. J Exp Clin Cancer Res. 2017;36(1):53. 28407783 10.1186/s13046-017-0528-y PMC5390430

[13] Yang SM , Huang C , Li XF , Yu MZ , He Y , Li J . miR‐21 confers cisplatin resistance in gastric cancer cells by regulating PTEN. Toxicology. 2013;306:162‐168. 23466500 10.1016/j.tox.2013.02.014

[14] Zhu W , Zhu DanXia , Lu S , et al. miR‐497 modulates multidrug resistance of human cancer cell lines by targeting BCL2. Med Oncol. 2012;29(1):384‐391. 21258880 10.1007/s12032-010-9797-4

[15] Zhang C , Liang Y , Ma MH , Wu KZ , Zhang CD , Dai DQ . Downregulation of microRNA‐376a in gastric cancer and association with poor prognosis. Cell Physiol Biochem. 2018;51(5):2010‐2018. 30522118 10.1159/000495820

[16] Esteller M . Cancer epigenetics for the 21st century: what's next? Genes Cancer. 2011;2(6):604‐606. 21941616 10.1177/1947601911423096 PMC3174266

[17] Liu H , Xu T , Cheng Y , et al. Altered 5‐hydroxymethylcytosine landscape in primary gastric adenocarcinoma. DNA Cell Biol. 2019;38(12):1460‐1469. 31657619 10.1089/dna.2019.4965

[18] Ito S , Shen LI , Dai Q , et al. Tet proteins can convert 5‐methylcytosine to 5‐formylcytosine and 5‐carboxylcytosine. Science. 2011;333(6047):1300‐1303. 21778364 10.1126/science.1210597 PMC3495246

[19] Tsai Y‐P , Chen H‐F , Chen S‐Y , et al. TET1 regulates hypoxia‐induced epithelial‐mesenchymal transition by acting as a co‐activator. Genome Biol. 2014;15(12):513. 25517638 10.1186/s13059-014-0513-0 PMC4253621

[20] Wang KC , Kang CH , Tsai CY , et al. Ten‐eleven translocation 1 dysfunction reduces 5‐hydroxymethylcytosine expression levels in gastric cancer cells. Oncol Lett. 2018;15(1):278‐284. 29285192 10.3892/ol.2017.7264 PMC5738697

[21] Orditura M , Galizia G , Sforza V , et al. Treatment of gastric cancer. World J Gastroenterol. 2014;20(7):1635‐1649. 24587643 10.3748/wjg.v20.i7.1635 PMC3930964

[22] Bang Y‐J , Van Cutsem E , Feyereislova A , et al. Trastuzumab in combination with chemotherapy versus chemotherapy alone for treatment of HER2‐positive advanced gastric or gastro‐oesophageal junction cancer (ToGA): a phase 3, open‐label, randomised controlled trial. Lancet. 2010;376(9742):687‐697. 20728210 10.1016/S0140-6736(10)61121-X

[23] Alsina M , Moehler M , Hierro C , Guardeno R , Tabernero J . Immunotherapy for gastric cancer: a focus on immune checkpoints. Targ Oncol. 2016;11(4):469‐477. 10.1007/s11523-016-0421-1 26880697

[24] Kamath SD , Kalyan A , Benson AB 3rd . Pembrolizumab for the treatment of gastric cancer. Expert Rev Anticancer Ther. 2018;18(12):1177‐1187. 30280940 10.1080/14737140.2018.1526084

[25] Ma F , An K , Li Y . Silencing of long non‐coding RNA‐HCG18 inhibits the tumorigenesis of gastric cancer through blocking PI3K/Akt pathway. OncoTargets Ther. 2020;13:2225‐2234. 10.2147/OTT.S240965 PMC7092690 32256081

[26] Zhou C , An N , Cao C , Wang G . lncRNA HOXC‐AS1 promotes gastric cancer via binding eIF4AIII by activating Wnt/beta‐Catenin signaling. J Gene Med. 2020:e3202. 32307743 10.1002/jgm.3202

[27] Luo M , Liang C . LncRNA LINC00483 promotes gastric cancer development through regulating MAPK1 expression by sponging miR‐490‐3p. Biol Res. 2020;53(1):14. 32293550 10.1186/s40659-020-00283-6 PMC7158027

[28] Dai Q , Zhang T , Pan J , Li C . LncRNA UCA1 promotes cisplatin resistance in gastric cancer via recruiting EZH2 and activating PI3K/AKT pathway. J Cancer. 2020;11(13):3882‐3892. 32328192 10.7150/jca.43446 PMC7171500

[29] Zhang F , Wang XS , Tang B , Li PA , Wen Y , Yu PW . Long non‐coding RNA FTX promotes gastric cancer progression by targeting miR‐215. Eur Rev Med Pharmacol Sci. 2020;24(6):3037‐3048. 32271421 10.26355/eurrev_202003_20668

[30] Dahl C , Gronbaek K , Guldberg P . Advances in DNA methylation: 5‐hydroxymethylcytosine revisited. Clin Chim Acta. 2011;412(11–12):831‐836. 21324307 10.1016/j.cca.2011.02.013

[31] Qu Y , Dang S , Hou P . Gene methylation in gastric cancer. Clin Chim Acta. 2013;424:53‐65. 23669186 10.1016/j.cca.2013.05.002

[32] Guo H , Zhu H , Zhang J , Wan B , Shen Z . TET1 suppresses colon cancer proliferation by impairing beta‐catenin signal pathway. J Cell Biochem. 2019;120(8):12559‐12565. 30825236 10.1002/jcb.28522

[33] Duan H , Yan Z , Chen W , et al. TET1 inhibits EMT of ovarian cancer cells through activating Wnt/beta‐catenin signaling inhibitors DKK1 and SFRP2. Gynecol Oncol. 2017;147(2):408‐417. 28851501 10.1016/j.ygyno.2017.08.010

[34] Wu J , Li H , Shi M , et al. TET1‐mediated DNA hydroxymethylation activates inhibitors of the Wnt/beta‐catenin signaling pathway to suppress EMT in pancreatic tumor cells. J Exp Clin Cancer Res. 2019;38(1):348. 31399111 10.1186/s13046-019-1334-5 PMC6688318

[35] Mo HY , An CH , Choi EJ , Yoo NJ , Lee SH . Somatic mutation and loss of expression of a candidate tumor suppressor gene TET3 in gastric and colorectal cancers. Pathol Res Pract. 2020;216(3):152759. 31859118 10.1016/j.prp.2019.152759

[36] Patel SP , Kurzrock R . PD‐L1 expression as a predictive biomarker in cancer immunotherapy. Mol Cancer Ther. 2015;14(4):847‐856. 25695955 10.1158/1535-7163.MCT-14-0983

[37] Ruf M , Moch H , Schraml P . PD‐L1 expression is regulated by hypoxia inducible factor in clear cell renal cell carcinoma. Int J Cancer. 2016;139(2):396‐403. 26945902 10.1002/ijc.30077

[38] Noman MZ , Desantis G , Janji B , et al. PD‐L1 is a novel direct target of HIF‐1alpha, and its blockade under hypoxia enhanced MDSC‐mediated T cell activation. J Exp Med. 2014;211(5):781‐790. 24778419 10.1084/jem.20131916 PMC4010891

